# Immunomodulatory Effect of a Cysteine-Rich Secretory Protein from an Entomopathogenic Nematode with Sterol-Binding Activity

**DOI:** 10.3390/toxins17070342

**Published:** 2025-07-05

**Authors:** Jorge Frias, Duarte Toubarro, Tiago Paiva, Nelson Simões

**Affiliations:** Biotechnology Centre of Azores (CBA), Faculty of Sciences and Technology, University of the Azores, 9500-321 Ponta Delgada, Açores, Portugal; duarte.nt.tiago@uac.pt (D.T.); tiago.m.paiva@uac.pt (T.P.); nelson.jo.simoes@uac.pt (N.S.)

**Keywords:** entomopathogenic nematodes, venom protein, immunomodulatory, sterol-binding, phenoloxidase, antimicrobial response

## Abstract

The *Steinernema carpocapsae* nematode is known to release several excretory/secretory products (ESPs) in its venom upon contact and during the parasitic infection process of insect hosts. A recurrent family of proteins found in this nematode’s venom is the CAP (cysteine-rich secretory protein/antigen 5/pathogenesis-related 1) protein, but the functional role of these proteins remains unknown. To elucidate the biological function, this study focused on characterising the secreted protein, first identified in the venom of the nematode’s parasitic stage, and the sequence retrieved from transcriptomic analysis. The structural comparisons of the Sc-CAP protein model, as determined by AlphaFold2, revealed related structures from other parasitic nematodes of vertebrates. Some of these closely related proteins are reported to have sterol-binding ability. The Sc-CAP recombinant protein was successfully produced in *Escherichia coli* in conjunction with a chaperone protein. The results showed that the Sc-CAP protein binds to cholesterol, and docking analyses of sterols on the protein revealed potential molecular interactions. Immunoassays performed in *Galleria mellonella* larvae revealed that this venom protein has an inhibitory effect against phenoloxidase and the antimicrobial response of insects. This suggests that the venom protein has an immunomodulatory function against insects, emphasising its importance during the parasite–host interaction.

## 1. Introduction

The control of insect pests in agriculture presents significant challenges, including the development of pest resistance and the adverse effects of chemical insecticides on beneficial species, human health, and the environment. Currently, there is still an intensive use of chemical insecticides to control various arthropod pests that have significant agricultural and health implications.

The need to find efficient alternatives to toxic chemical insecticides has drawn the attention of the scientific community to biological control agents (BCAs), such as entomopathogenic bacteria, fungi, and nematodes, which are effective in combating a wide variety of insect pests.

Entomopathogenic nematodes (EPNs) have been used as an effective BCA and as an alternative for more sustainable and safe pest control in agriculture. Lately, the use of these BCAs has shown some limitations as an alternative to chemical pesticides. These encompass some difficulties in their mass production and storage, as well as a limited understanding of the nematode strains that are more virulent against specific insects [1,2]. For this reason, the use of BCAs that include EPNs remains an area of intensive investigation by the scientific community to improve their virulence characterisation, mass production, and application in the agricultural field [3,4].

There is an enormous focus on two particular families of nematodes—*Heterorhabditidae* and *Steinernematidae*. The greater interest seen in these two mentioned families is mainly because they can be produced on a much more extensive (industrial) scale and used in the formulation of commercialised agricultural products [5].

Considering the well-characterised entomopathogenic nematode *Steinernema carpocapsae*, which is in symbiosis with the bacterium *Xenorhabdus nematophila*, it is known to form a highly virulent complex to insects. For this reason, it is widely used as a BCA in agriculture [6].

The virulence of this EPN is linked not only to its symbiotic bacteria but also to its capacity to produce molecular effectors [7,8]. This nematode is known to release several excretory/secretory products (ESPs) upon contact and during the parasitic infection process of its hosts [9]. There is a continuous need to characterise virulence factors to enhance the understanding of host–pathogen interactions and better differentiate between various EPN strains based on these effectors.

Different proteins have been identified in the nematode ESPs, some of which are immunomodulators, and others cause tissue damage and insect lethality [10,11,12,13].

Several enzymes are also reported to be released, causing tissue damage and inhibition of different important defence systems, such as the insect clotting mechanism [14] and phenoloxidase system [15]. The most abundant groups of proteins present in ESPs are serine proteases and metallopeptidases, but other virulence factors that exist in less abundance may contribute collectively to insect toxicity and immune suppression, including other less-represented protein families such as retinol-binding proteins (FAR), ShK-like proteins, and venom allergen-like proteins (VAL), also known as cysteine-rich secretory protein/antigen 5/pathogenesis-related 1 proteins (CAP) [13,16,17,18].

One recurrent yet uncharacterised protein family identified in the ESPs of *S. carpocapsae* is the CAP protein family. These proteins in other organisms have been involved in different biological processes such as reproduction, fungal virulence, cellular defence, immune evasion, and pathogen defence, and in plants, they have sterol-binding capability, which is important for protection against fungal pathogen oomycetes [19]. This superfamily of proteins has been successively identified in several other nematode parasites of vertebrates, including *Brugia malayi*, *Necator americanus*, *Ancylostoma caninum*, and *Heligmosomoides polygyrus*. Several studies have reported that this family of proteins, released by parasitic nematodes, has sterol-binding ability; however, the functional role of these proteins in their respective hosts, as well as the mode of action, remains unknown [20,21,22].

In this study, we cloned, expressed, and characterised a CAP protein from *S. carpocapsae* (Sc-CAP), which was first identified in the ESPs from the parasitic stage of the nematodes induced with insect tissue homogenate, and the sequence was retrieved from transcriptomic analysis [23].

This study demonstrates for the first time that the Sc-CAP protein binds to signalling lipid compounds and has an immunosuppressive effect on critical defence systems in the insect host.

## 2. Results

### 2.1. Optimisation and Heterologous Expression of the Recombinant Protein

**Figure 1:**
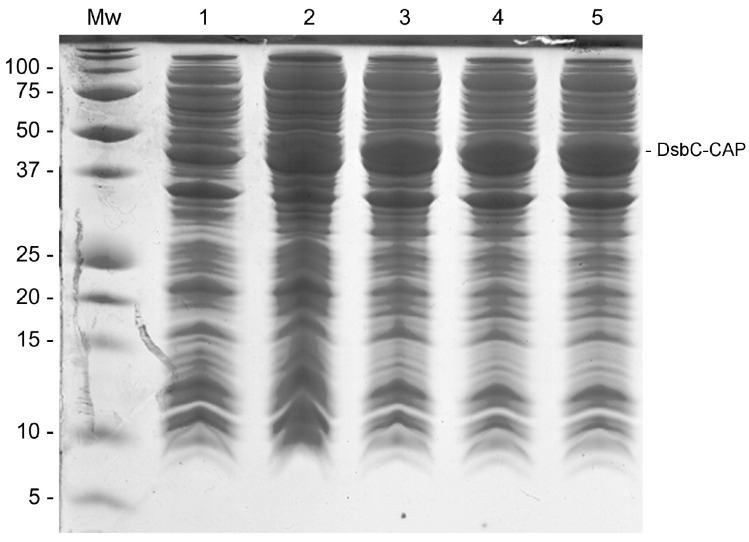
The SDS-PAGE gel displaying different production conditions of DsbC-CAP in *E. coli*. Lane Mw: Protein molecular weight standard. Lane 1: Clarified lysate of non-induced cells incubated at 37 °C and 250 rpm for 3 h. Lanes 2–5: Cells were initially incubated at 37 °C and 250 rpm for 3 h. Lane 2: Clarified lysate of induced cells grown further at 28 °C for 6 h. Lane 3: Clarified lysate of induced cells grown further at 28 °C overnight. Lane 4: Clarified lysate of induced cells grown further at 25 °C for 24 h. Lane 5: Clarified lysate of induced cells grown further at 28 °C for 24 h.

In order to increase the chances of Sc-CAP protein expression, which is a cysteine-rich protein, the pHTP4 expression vector containing a disulfide-bond isomerase C (DsbC) fusion protein was used to clone the gene encoding the venom protein from *S. carpocapsae*. This isomerase assists in the proper folding of proteins by rearranging disulfide bonds [24]. The DsbC-CAP recombinant protein was expressed in *E. coli* RII (DE3) under different growing conditions in an auto-induction medium, and the clarified lysate was analysed by gel electrophoresis (Figure 1). No overexpression protein band was observed in the protein profile of the cells incubated at 37 °C and 250 rpm for three hours (Figure 1, lane 1). In this condition, bacteria were in a non-induced state while consuming glucose from the medium.

When bacteria were allowed to grow beyond three hours of incubation, the medium became depleted of glucose, and the consequent consumption of lactose induced the heterologous expression of recombinant protein in the cells. This was evident in the other four experimental growing conditions (Figure 1, lanes 2–5), where an overexpressed target band with the expected molecular weight of 50 kDa was observed in every indicated lane. The optimal growth conditions for the expression of DsbC-CAP were revealed to be at 28 °C and 250 rpm overnight (Figure 1, lane 3), with similar results for the other two subsequent experimental conditions at 25 °C for 24 h and at 28 °C for 24 h (Figure 1, lanes 4 and 5). Cells incubated at 28 °C for 6 h (Figure 1, lane 2) presented a lower expression of recombinant protein compared with the other three conditions, revealing the importance of incubation time for protein expression in this case. These optimal conditions were used in the scale-up production of the recombinant protein for the purification step.

### 2.2. Purification of the Recombinant Protein

**Figure 2:**
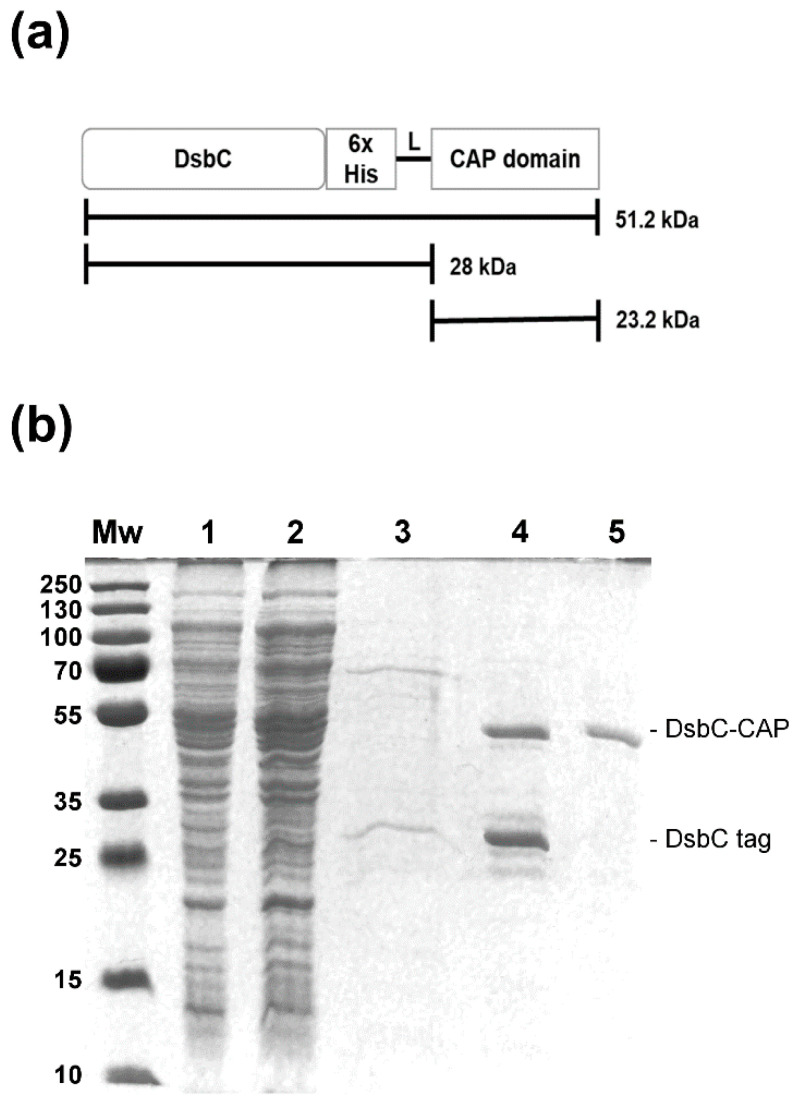
Recombinant protein purification by nickel–nitrilotriacetic acid affinity chromatography (Ni-NTA). (**a**) Full scheme of the recombinant protein, including the DsbC, Sc-CAP domain, and polyhistidine tag. (**b**) SDS-PAGE gel demonstrating the production of the DsbC-CAP recombinant protein in *E. coli* RII (DE3) and the various steps of the purification protocol. Lane Mw: Protein molecular weight standard. Lane 1: Soluble lysate following the sonication and centrifugation of induced cells containing the expression vector. Lane 2: Ni-NTA column flowthrough. Lane 3: Ni-NTA column wash with 50 mM imidazole. Lane 4: Eluate from the Ni-NTA agarose column of recombinant protein. Lane 5: Protein dialysis against PBS using a centrifugal membrane filter with a 50 kDa cutoff.

The Sc-CAP encoding sequence was inserted into the C-terminal region of periplasmic DsbC along with a 6xHis-tag (Figure 2a). The DsbC-CAP fusion protein was expressed under the previously determined optimal conditions and purified from the clarified crude extract (soluble fraction) by nickel affinity chromatography, and protein profiles were analysed by SDS-PAGE (Figure 2b).

Analysis of the elution fraction revealed a double-band protein corresponding to the DsbC-CAP fusion protein with an expected molecular size of 50 kDa and a low molecular weight band corresponding to the truncated version composed of the DsbC tag of approximately 28 kDa in molecular size (Figure 2b, lane 4). High purity and yield of the recombinant protein (20 mg/L) were achieved after being dialysed using a centrifugal membrane filter with a 50 kDa cutoff to remove DsbC truncated forms. Electrophoresis analysis was performed to confirm the presence of fusion protein species (Figure 2b, lane 5).

LC-MS analysis of the protein corresponding to the excised band yielded 37 peptides (Supplementary Table S1). The total length of peptides covers 42.3% of the full protein sequence matching the DsbC-CAP recombinant protein, with a significant value of 101 (values above 61 are considered significant at a *p*-value of less than 0.05). A second match with an SCP domain-containing protein (acc. nº L596_010601) was found when searched against the UniProt protein sequence database with a taxonomy restriction to *Steinernema*. Further Blast searches of the Sc-CAP sequence revealed an exact match with a cysteine-rich protein (acc. nº L596_g15954.t1, found in supplementary material of Lu et al. (2017) [16]), which was previously detected in the ESPs of *Steinernema carpocapsae*.

### 2.3. Tertiary Structure Prediction of the Sc-CAP Protein and Comparison to Other Closely Related Structures

The tertiary structure of the Sc-CAP protein was predicted by AlphaFold2 ColabFold v1.5.5 with a per-residue estimated model confidence of pLDDT > 90 between positions 30–215, indicating it was modelled with high accuracy (Supplementary Figure S1).

**Figure 3:**
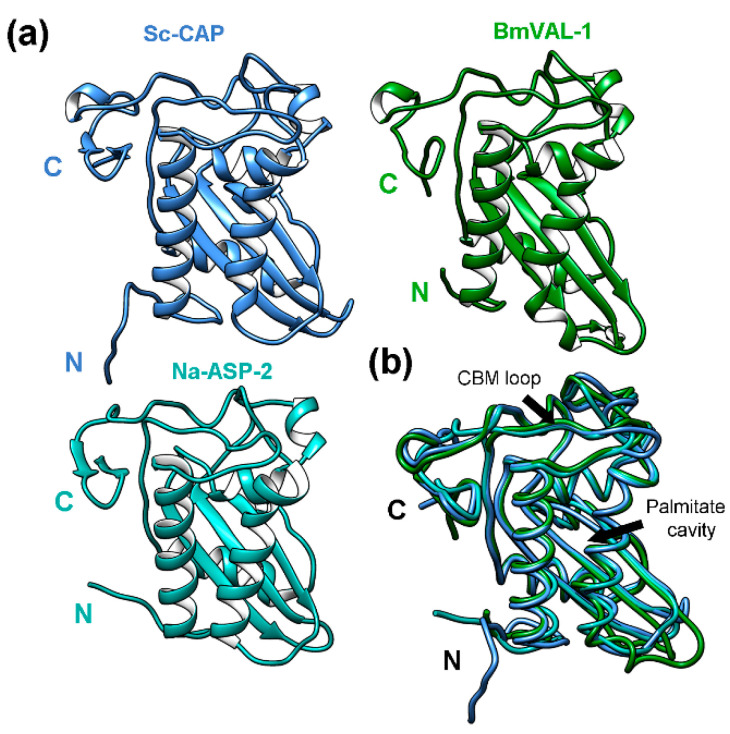
The 3D structure of the Sc-CAP protein and its similarities to other proteins from parasitic nematodes. (**a**) The Sc-CAP protein model predicted by AlphaFold2 ColabFold v1.5.5 software and closely related structures: BmVAL-1 (6ANY) from *Brugia malayi* and Na-ASP-2 (4NUK) from *Necator americanus*. (**b**) The superimposition of the Sc-CAP 3D model (blue) backbone with the closest-to-average structures BmVAL-1 (green) and Na-ASP-2 (cyan). The longest helices forming the palmitate cavity and the caveolin binding motif loop (CBM) are indicated with arrows.

The Sc-CAP domain of 216 amino acids adopts a unique alpha-beta-alpha sandwich typical of CAP superfamily proteins [19], as observed in Figure 3a.

The multiple structural comparisons of the Sc-CAP AlphaFold-generated model using the DALI server against the Protein Data Bank (RCSB PDB) revealed several related structures from other parasitic nematodes of vertebrates (Table 1).

The identified homolog protein with the highest Z-score recorded was BmVAL-1 (6ANY) from *Brugia malayi*. Despite this protein showing a sequence identity of 43%, the structure presents a high number of superimposed residues (Lali = 199) and a good average (RMSD = 1.7) distance between superposed atoms relative to the Sc-CAP protein. Two other closely related structures from parasitic nematodes were identified: a Na-ASP-1 domain (3NT8) with a Z-score of 28.2 and a Na-ASP-2 protein (4NUK) with a Z-score of 28.5, both from *Necator americanus* and with unknown biological function. The structures with the lowest Z-score recorded were HpVAL-4 (5WEE) from *Heligmosomoides polygyrus bakeri* and HPI (4TPV) from *Ancylostoma caninum*. Among the identified proteins, only BmVAL-1 and HpVAL-4 have known biological functions. These were identified as sterol-binding proteins [22,25].

The superimposition of the Sc-CAP structure with the other two proteins, BmVAL-1 and Na-ASP-2, which had the highest Z-scores recorded (Figure 3b), revealed almost identical structures with slight variations between residues in the N-terminal region. Despite these proteins showing a sequence identity ranging from 41 to 43% relative to the Sc-CAP protein, the lengths and exact positions of secondary elements, such as alpha helices and beta sheets, are almost identical among the structures. All three structures adopt an alpha-beta-alpha sandwich typical of CAP superfamily proteins. Similarly to BmVAL-1, they adopt different sterol-binding cavities, such as the palmitate cavity between the two longest helices and the caveolin binding motif loop (CBM) close to the C-terminal region of the protein [25].

**Figure 4:**
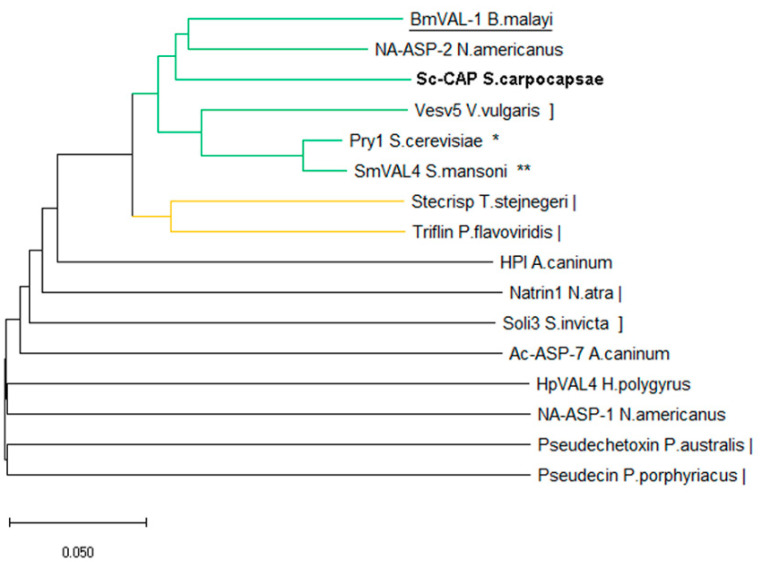
Structure-based phylogenetic tree of CAP proteins from nematodes and their relationship to other toxins from fungi (*), platyhelminthes (**), chordata (|), and arthropoda (]). The protein of interest in this study is shown in bold, and the closest structurally related protein is underlined. The Q-score distance matrix produced by PDBefold was utilised to compute a phylogenetic tree. The neighbour-joining method was used to infer the phylogeny. The ideal tree is displayed with a branch length sum of 2.09228027. Evolutionary analyses were carried out in MEGA X [26].

Results from the structure-based multiple comparison analysis suggest that Sc-CAP is phylogenetically distant from the CAP proteins of the phylum *Chordata* and is closely related to those of *Arthropoda*, *Platyhelminthes*, and *Fungi* (Figure 4).

The Sc-CAP protein was localised in the same branch as the BmVal-1 and Na-ASP-2 proteins, in accordance with the observed superposition of the structures. Another close branch is composed of Ves v5 from *Vespula vulgaris*, Pry1 from *Saccharomyces cerevisiae*, and SmVAL4 from *Schistosoma mansoni*. The other branches are primarily composed of proteins from the phylum Chordata, along with a few structures belonging to nematodes.

### 2.4. In Silico Interaction of Sterols with the Sc-CAP Protein

**Figure 5:**
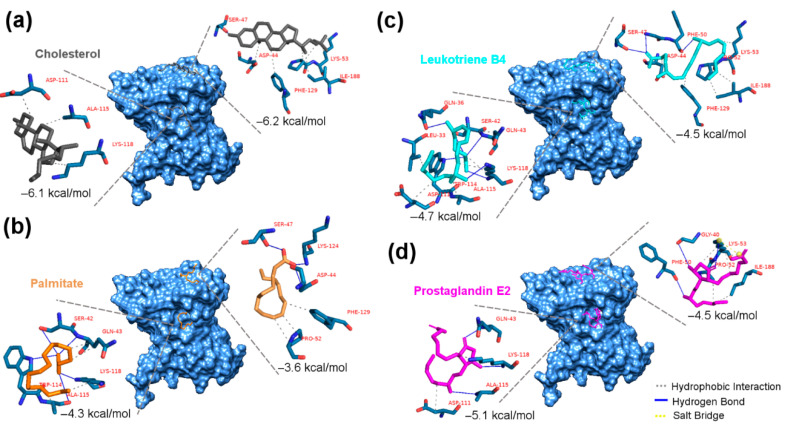
Predicted docking poses of sterols on the Sc-CAP protein. Top-scoring docking poses are shown for (**a**) cholesterol (black-grey), (**b**) palmitate (orange), (**c**) leukotriene B4 (cyan), and (**d**) prostaglandin E2 (magenta), docked at both the CBM and PBC binding pockets of the Sc-CAP protein. The residues that are highlighted in blue and the legend in red represent the interacting amino acids from each binding pocket. Hydrophobic interactions are depicted as grey dashed lines, hydrogen bonds as solid blue lines, and salt bridges as yellow dashed lines.

Docking analysis of cholesterol on the Sc-CAP protein resulted in all 10 top-scoring poses with cholesterol located in the expected caveolin motif loop (CBM) and forming a hydrophobic interaction with five amino acids (Lys-53, Ile-188, Phe-129, Asp-44, and Ser-47) that are part of the CBM. The top-scoring pose of cholesterol on the Sc-CAP has an energy binding of −6.2 kcal/mol, indicating a slightly higher affinity towards the CBM than to the PBC, where it forms only three hydrophobic interactions (Figure 5a).

In the case of palmitate, the interactions with binding motifs were opposite to those of cholesterol. This molecule interacted with higher affinity in the cavity between two alpha helices of the protein, with an energy of binding of −4.3 kcal/mol. In contrast, it had a lower affinity to the CBM (−3.6 kcal/mol). It presented five hydrogen bonds with amino acids Ser-42, Trp-114, Ala-115, and Lys-118 and three hydrophobic interactions with Gln-43 and Lys-118 in the expected PBC (Figure 5b).

Both sterols interacted differently with the Sc-CAP protein in terms of affinity and binding sites. Cholesterol interacted with a higher affinity than palmitate, as indicated by the obtained energy binding results.

Regarding the docking results of both eicosanoids, they interacted with slightly better affinity towards the PBC, forming more molecular hydrophobic and hydrogen bonds. The leukotriene B4 interacted with the CBM, exhibiting a binding energy of −4.5 kcal/mol, while displaying an energy binding of −4.7 kcal/mol with the PBC (Figure 5c). It formed two hydrogen bonds with an additional Phe-50 amino acid, unlike cholesterol in the same CBM binding pocket, and presented more interactions than palmitate in the alternative pocket, forming hydrogen and hydrophobic bonds with three additional amino acids (Gln-36, Leu-33, and Asp-111).

In the case of prostaglandin E2, it interacted with distinct amino acids in the CBM site, sharing only the amino acids Lys-53 and Ile-188 with cholesterol in the same pocket, resulting in a lower energy binding of −4.5 kcal/mol. This sterol interacted more effectively in the PBC with an energy binding of −5.1 kcal/mol, despite having fewer molecular interactions with the same amino acids as palmitate, suggesting shorter distances between the ligand and receptor (Figure 5d).

### 2.5. In Vitro Sterol-Binding Ability of the Sc-CAP Protein

**Figure 6:**
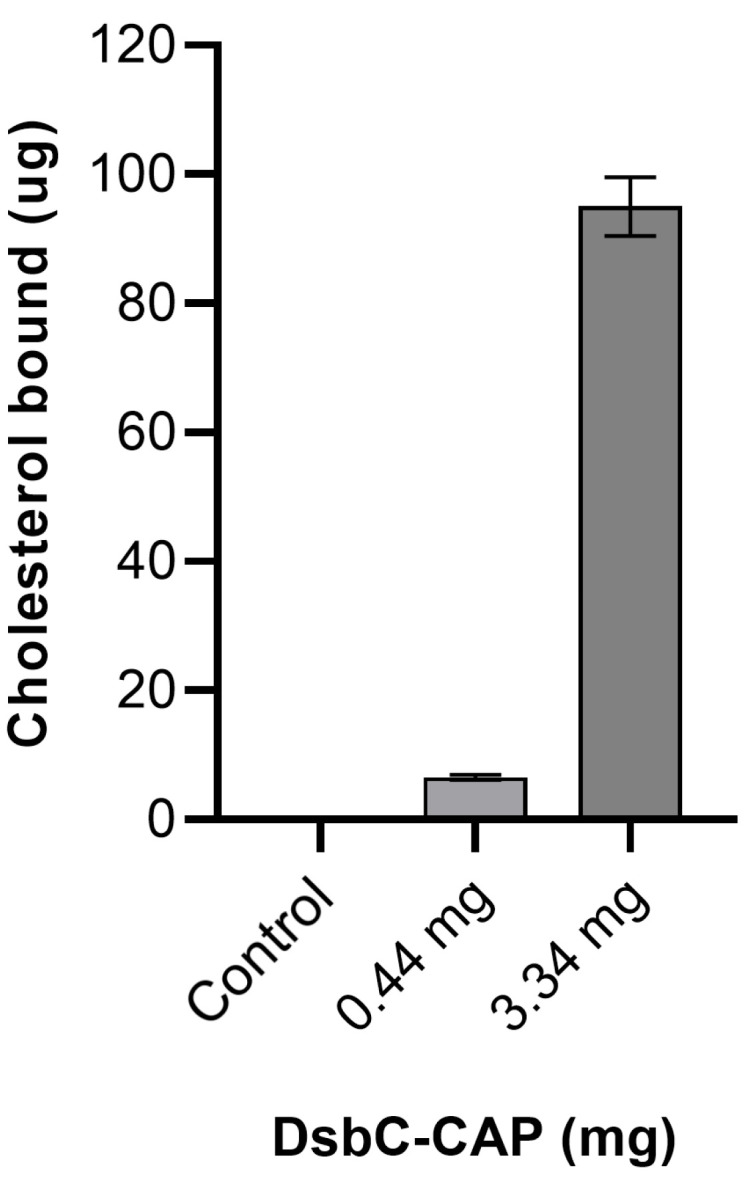
In vitro sterol-binding assay of cysteine-rich secretory protein/antigen 5/pathogenesis-related 1 (Sc-CAP) from *Steinernema carpocapsae*. The recombinant Sc-CAP was assessed for cholesterol-binding activity using the nickel affinity chromatography co-purification method. As a negative control, the DsbC tag alone (without Sc-CAP) was tested in parallel under identical conditions in PBS to assess background signal and nonspecific binding. Data are presented as mean ± S.D. (*n* = 9).

During the in vitro cholesterol-binding assay, the addition of different amounts of protein resulted in the concentration-dependent binding of cholesterol to the recombinant protein Sc-CAP (Figure 6).

Recombinant proteins at a low concentration of 0.44 mg/mL, when incubated with 80 μg/mL of cholesterol, demonstrated the ability to bind and sequester approximately 6.5 μg of cholesterol. A 7-fold increase in the amount of recombinant protein resulted in approximately a 15-fold increase in cholesterol sequestration, reaching up to 95 μg of cholesterol. These results demonstrate that Sc-CAP binds to cholesterol in a concentration-dependent manner, supporting the in silico docking results.

### 2.6. Phenoloxidase Inhibition Effect of the Sc-CAP Protein

**Figure 7:**
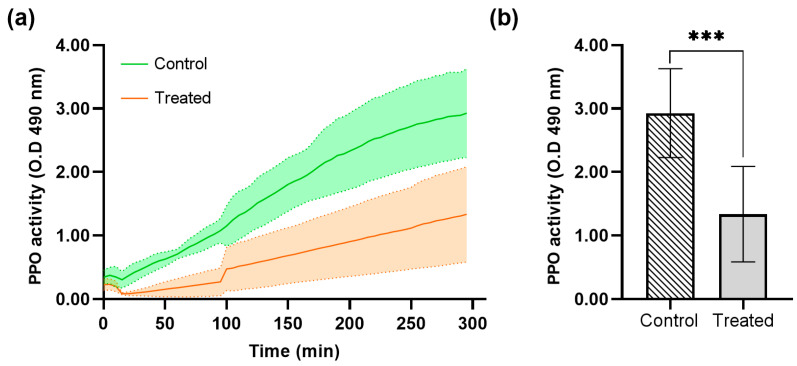
Prophenoloxidase activity (PPO) and optical density (OD) average at 490 nm in the *Galleria mellonella* larvae hemolymph treated with recombinant protein DsbC-CAP. (**a**) Phenoloxidase activity kinetics over 300 min for the control (*n* = 12) and treated (*n* = 12) groups. (**b**) Maximum phenoloxidase activity was achieved in the control and treated groups. Data are presented as mean ± S.D. The asterisks (***) show a significant difference (*p* < 0.001) between the control and treated groups (unpaired *t*-test).

The prophenoloxidase (PPO) activity measured in the hemolymph of *Galleria mellonella* larvae treated with recombinant protein DsbC-CAP was compared to the control group injected with DsbC tag in PBS, as depicted in Figure 7.

The control group, which was not treated with the DsbC-CAP recombinant protein, showed a normal level of PPO activity (Figure 7a). This serves as the reference point for evaluating the effects of the Sc-CAP venom protein. The treated group with Sc-CAP exhibited a significant decrease in PPO activity compared to the control group, as confirmed by statistical analysis using an unpaired t-test (*p* < 0.001), as shown in Figure 7b. These results demonstrate that larvae treated with the venom protein exhibited a slower phenoloxidase response, reaching only half of their maximum potential prophenoloxidase activity.

### 2.7. Hemolymph Antimicrobial Response

**Figure 8:**
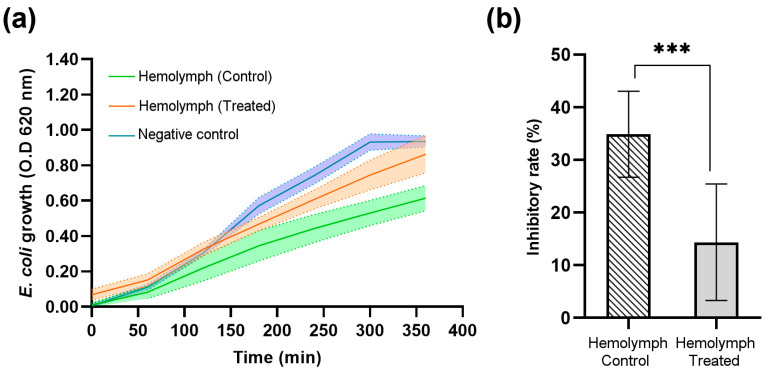
*Escherichia coli* bacterial growth and optical density (OD) average at 620 nm in the *Galleria mellonella* larvae hemolymph treated with recombinant protein (DsbC-CAP) and treated with DsbC tag in PBS (hemolymph control). The negative control group consisted of bacterial growth in LB medium only. (**a**) Bacterial growth curves over 350 min for the control (*n* = 11), treated (*n* = 10), and negative control groups (*n* = 10). (**b**) The maximum inhibition rate of bacterial growth achieved by the hemolymph-treated and control groups. The inhibition rates were calculated based on the equation described by Guo et al. (2025) [27]. Data are presented as mean ± S.D. The asterisks (***) show a significant difference (*p* < 0.001) between the control and treated groups (unpaired *t*-test).

The antimicrobial activity of *G. mellonella* larvae hemolymph treated with the recombinant protein DsbC-CAP was assessed by monitoring the growth of *E. coli* over time and comparing it to the controls (Figure 8).

Three experimental groups were evaluated in a time-course analysis of *E. coli* growth (Figure 8a). The negative control group, which represents bacterial growth without the presence of insect hemolymph, was used to obtain a normal bacterial growth curve, reaching an optical density (OD) of approximately 1.0 at 620 nm by 350 min. The hemolymph-treated group, which represents bacterial growth in the presence of hemolymph from treated insects with the recombinant protein DsbC-CAP, shows that while bacterial growth was slightly inhibited, it still increased over time, being close to the OD reached by the negative control at the end of the experiment. Finally, the hemolymph control group, representing bacterial growth in the presence of hemolymph from treated insects with the DsbC tag only, showed the greatest inhibition of bacterial growth. The OD remained consistently lower than the other two groups, with a final OD below 0.6 at 350 min, indicating a stronger antimicrobial response than the negative control and hemolymph-treated groups.

The endpoint analysis of *E. coli* growth (Figure 8b) further highlights the differences in inhibitory rates achieved by hemolymph of both control and treated insect groups. The hemolymph CAP-treated group showed slightly reduced bacterial growth (OD ~ 0.9) compared to the negative control group (OD ~ 1.0), but this difference was not statistically significant (NS). This indicates that CAP-treated insects have significantly impaired antimicrobial defences against Gram-negative bacteria, resulting in a lower inhibition rate of bacterial growth (14%). Furthermore, the DsbC tag-treated hemolymph (control group) exhibited a significantly higher inhibition rate of bacterial growth (35%), as indicated by the asterisks (***), denoting a highly significant difference (*p* < 0.001). The inhibition of bacterial growth in the hemolymph of DsbC tag-treated insects (control group) represents the maximum expected under specified growth conditions.

## 3. Discussion

There is no doubt that entomopathogenic nematodes, such as those in the *Heterorhabditidae* and *Steinernematidae* families, constitute a rich source of different types of molecular effectors to explore. During the nematode infection state, they release a wide range of ESPs with diverse biological functions to interact with their host [9,15]. Most of them are immunomodulators that help the nematode surpass the defence mechanisms of insects.

The CAP family is one of the unexplored proteins present in the ESPs of *S. carpocapsae*, which may play a crucial role during the infection stage of the nematode. Members of this family of proteins, also known as venom allergen-like proteins (VAL), were extensively studied in several other parasitic nematodes of vertebrates [28]. This family presents high expression levels in many parasitic nematodes and is a potential vaccine candidate because of their immunogenic properties. These CAP proteins are homologs of vespid (wasp) venom proteins known to cause allergic, toxic, and inflammatory responses in humans [25].

In this study, heterologous expression and characterisation of the *S. carpocapsae* recombinant protein with homology to the CAP superfamily were achieved.

This Sc-CAP is a cysteine-rich protein that contains ten cysteines and is expected to form five disulfide bonds. Generally, the recombinant expression of cysteine-rich proteins that form multiple disulfide bonds in the bacterium *E. coli* is challenging due to the lack of effective post-translational processing in these systems. In the case of Sc-CAP, to increase the chances of heterologous expression in *E. coli*, the periplasmic DsbC was fused to the N-terminal of the venom protein. Several studies have reported the DsbC tag as a highly efficient partner for the recombinant expression of disulfide-rich proteins in *E. coli* [24,29,30].

A high production yield of the DsbC-CAP recombinant protein was achieved in the soluble fraction of *E. coli* clarified lysate. Interestingly, during the optimisation of recombinant protein expression, the increase in incubation time had a greater impact than the variation in temperature during bacterial growth. After purification of the recombinant protein, the presence of truncated forms of the fusion protein (DsbC fusion tag) was verified during the electrophoresis analysis of elution fractions. An overexpressed heavier band of approximately 50 kDa, representing more than 50% of the total eluted protein that corresponds to the DsbC-CAP fusion species, was detected in every condition during the optimisation experiments. Due to the size difference between the DsbC tag and the fusion protein species, purification of the full recombinant protein version was successfully achieved using a membrane with a 50 kDa cutoff. This process resulted in obtaining DsbC-CAP in a soluble and active form, as demonstrated by both in vitro and in vivo assays.

The determination of the Sc-CAP tertiary structure using the artificial intelligence algorithm AlphaFold2 ColabFold v1.5.5, along with multiple structural comparisons, enabled the identification of protein homologs from other parasitic nematodes of vertebrates. Despite the Sc-CAP sequence revealing low sequence identity (<50%) with closely related proteins, the predicted structure highly resembles that of BmVAL-1 from *Brugia malayi* and Na-ASP-2 from *Necator americanus*.

One study reported the structure and function of BmVAL-1, and it found three distinct and separate cavities in the structure: the palmitate cavity, localised between two long alpha-helices; the caveolin-binding motif (CBM); and the central cavity, which is essential for the sterol-binding ability of the protein. Additionally, they reported the sterol-binding ability of BmVAL-1 using both in vivo and in vitro assays [25]. Another study reported a highly similar structure, Hp-VAL-4 from *H. polygyrus*, with the same sterol-binding cavities. They also demonstrated the recombinant protein’s ability to bind to cholesterol by in vitro and in vivo assays [22].

Cholesterol plays a vital role in insect defence mechanisms, primarily as a precursor for steroid hormones, particularly ecdysone, which is essential for insect development and immunity. Insects cannot synthesise cholesterol themselves and rely on dietary sources or symbiotic microorganisms for this crucial compound. A sufficient supply of cholesterol is necessary in the diet to support the immune system during infections. This compound is a precursor to steroid hormones, with ecdysone being the most important for the immune system (in its active form, β-ecdysone), which has been found to regulate the PPO system in *Locusta migratoria* [31,32].

For this reason, the ability of the Sc-CAP protein to bind to sterols was assessed by in silico and in vitro assays. The molecular docking results demonstrate that Sc-CAP can structurally bind and sequester sterols from the environment in which they are secreted, including eicosanoids, a group of biologically active lipid molecules that play important roles in regulating several insect physiological processes, such as inflammation, immune responses, and coagulation [33,34]. This was further confirmed and supported by the in vitro cholesterol assay, which showed the cholesterol-binding ability of the recombinant protein.

These results provide evidence that Sc-CAP can structurally bind to sterols. Many fatty acids and fatty acid-derived products have a broad spectrum of biological roles in insect physiology, including reproduction, fluid secretion, hormone actions, and immunological relevance, such as prostaglandins and leukotrienes [33].

Interestingly, both leukotriene B4 and prostaglandin E2 interacted with the same amino acids as cholesterol and palmitate in identified pockets of the protein and even presented in some cases hydrophobic and hydrogen bonds with additional amino acids, indicating more specificity and moderate affinity (between −4.5 and −5.1 kcal/mol). Thus, this suggests that this venom protein can bind to these compounds, which may have an immunomodulatory function against insects, assisting in the camouflage of the nematode during invasion of the insect host. Other studies also hypothesised that the presence of fatty acid- and retinol-binding proteins (FARs) on the nematode ESPs could be related to the suppression of host immunity during nematode infection [35].

In this context, the proteins of the CAP superfamily are possible candidate genes associated with nematode virulence. Significant research has been conducted on this family, particularly due to their high expression in many parasitic nematode species. The precise biological role of CAP proteins during infection remains to be determined; however, since they are secreted sterol-binding proteins, there is a good possibility that they will interact with immunomodulating molecules that stimulate and regulate immune response elements [28].

In this work, we show the inhibitory effect of Sc-CAP protein in the phenoloxidase system and antimicrobial peptide production of treated insects, thus suggesting that it has an important role in immunosuppressing these major defence systems of the insect host during the nematode invasion and infection process. This effect has already been observed in other studies using the total venom and cuticles of entomopathogenic nematodes in various insect hosts [9,15,36,37,38].

Some molecular effectors with phenoloxidase inhibitory effects have already been reported from *S. carpocapsae* ESPs, suggesting that different types of proteins participate in this process and help the nematode evade this major insect defence system [11,39,40]. A deeper understanding of virulence factors with immunosuppressing activity is crucial for comprehending the molecular mechanisms of nematode pathogenicity and as a source of discovery for new molecules with therapeutic applications for autoimmune diseases.

The focus of this work was to characterise the function of the Sc-CAP protein from *S. carpocapsae*; however, future work is necessary to evaluate the mode of action by examining the molecular-level response of treated insects. The effectiveness of this venom protein in combination with different biological control agents against insects is a factor to be considered to show the relevance of this venom protein during the infection process. The competitive binding ability of Sc-CAP to sterols could constitute a novel approach, complementing other insect control strategies and demonstrating its potential use as an immunosuppressing molecule or as a candidate gene for the virulence improvement of entomopathogenic nematode strains used in biological control.

## 4. Conclusions

This study’s findings suggest that the Sc-CAP protein plays a crucial role during the parasitic infection process: it acts as an immunomodulator to suppress the insect’s defence responses and as a lipid-binding agent, possibly interfering with the host’s immune signalling processes. This immunosuppression activity may be linked to the sterol-binding capability of this venom protein, as many fatty acids and fatty acid-derived products, such as prostaglandins and leukotrienes, play important roles in the insect immune system.

Our study provides significant insights into the functional roles of CAP proteins in the venom of *S. carpocapsae*, emphasising their importance in the parasite–host interaction. This work lays the groundwork for future research on the mode of action of this protein and further explores its potential as a target for developing novel biocontrol strategies or improving existing biocontrol agents, thereby contributing to the sustainable management of insect pests in agriculture.

## 5. Materials and Methods

### 5.1. Materials

Molecular biology reagents were ordered from Thermo Fisher Scientific (Lisbon, Portugal), except the NZYEasy Cloning kit IV, which includes the linearised pHTP4 vector sourced from NZYTech genes and enzymes (Lisbon, Portugal). All other chemicals and reagents were from Sigma-Aldrich (Lisbon, Portugal), including the CelLyptic^TM^ B Cell Lysis Reagent (B7435), His-Select Nickel Magnetic Agarose Beads (H9914), and cholesterol quantification assay kit (CS0005). The gene that encodes the Sc-CAP protein (NCBI acc. nº ON088290) was synthesised de novo with optimised codons for expression in *E. coli*. This was achieved using the ATGenium codon optimisation algorithm. The gene was delivered in the pUC57 vector by NZYTech Genes and Enzymes (Lisbon, Portugal), located in Portugal. The primers used in the cloning process were created by STAB VIDA (Setubal, Portugal).

### 5.2. Expression Vector Construction

To construct the pHTP4/CAP expression vector, the DNA sequence encoding the gene Sc-CAP was amplified from the pUC57 vector by PCR using two overlapping gene-specific primers (Fwd: 5′-tcagcaagggctgaggATGCAGGAAGATCCGGC-3′; Rev: 5′-tcagcggaagctgaggGTTTTTAATGCACAGGCCTTC-3′), both containing non-priming overhangs complementary to the linearised pHTP4 vector. The parental plasmid was removed from the PCR product using DpnI by incubating it for one hour at 37 °C and then heat-inactivating it for twenty minutes at 80 °C. The purified PCR product was cloned into the pHTP4 expression vector using the NZYEasy cloning kit (Lisbon, Portugal). The PCR product was mixed with 100 ng of linearised vector, using a molar ratio of 1:5 (vector/insert). The cloning reaction was performed in a 10 μL volume in a PCR tube and incubated for 1 h at 37 °C on a heating block. The mixture was then incubated at 80 °C for 10 min, followed by 10 min at 30 °C. The pHTP4 expression vector, containing the CAP protein gene, was transformed into chemically competent *E. coli* TOP10 cells by thermal shock. Briefly, 1 µL of the cloning reaction mix was added to 50 µL of the bacterial suspension and incubated on ice for 30 min. This was followed by a heat shock at 42 °C for 30 s, and then the sample was chilled on ice for 5 min. Transformed bacteria were recovered in 1 mL of rich broth medium for 1 h at 37 °C and plated on an LB plate containing 50 µg/mL of kanamycin and incubated overnight. Colony PCR was performed by screening a maximum of 10 colonies using primers to amplify the insert region of the pHTP4 vector (Fwd: 5′-CAATGGCACACTTGTTCCGGGTTAC-3′; Rev: 5′-GGTTATGCTAGTTATTGCTCAGCG-3′). The PCR reaction was performed under the following conditions: 95 °C for 20 min, followed by 30 cycles of 95 °C for 50 s, 55 °C for 1 min, and 72 °C for 1 min, with a final extension at 72 °C for 5 min. The plasmids from positive colonies were purified using a miniprep kit (K0503), and the DNA concentration was determined by NanoDrop (Thermo Fisher Scientific, Lisbon, Portugal).

### 5.3. Optimisation and Heterologous Expression of DsbC-CAP in E. coli

For expression analysis, two batches of *E. coli* Rosetta II (DE3) cells were transformed with the pHTP4/CAP plasmid and the empty pHTP4 plasmid, respectively, as described previously. The transformed cells were cultured in 5 mL of LB liquid medium with the addition of 50 µg/mL of kanamycin and grown overnight at 37 °C. Small batches of 50 mL of ZYP-5052 auto-induction medium (2% tryptone, 0.5% yeast extract, 0.5% NaCl, 0.5% glycerol, 0.05% glucose, and 0.2% lactose in phosphate buffer, pH 7.2) supplemented with 50 µg/mL of kanamycin as well as 1.25 mL of starter culture were used to optimise the expression conditions. In total, five different growth conditions were tested: one culture batch was incubated by shaking at 37 °C and 250 rpm for 3 h, and then the cells were harvested. The other four batches were initially incubated with shaking at 37 °C and 250 rpm for 3 h and then allowed to grow at different incubation temperatures and times: 6 h at 28 °C, overnight at 28 °C, 24 h at 25 °C, and finally 24 h at 28 °C.

All cell batches were collected by centrifugation at 4500 rpm for 30 min at 4 °C. Pellets were washed with 0.8% NaCl solution and frozen at −80 °C for 1 h. Next, the cell pellets were thawed and lysed at 4 °C for 30 min in 20 mM phosphate buffer saline, pH 7.4 (PBS), by the addition of 10× concentrated CelLyptic^TM^ B Cell Lysis Reagent supplemented with lysozyme to a final concentration of 300 µg/mL and 1 µL DNase solution (1 mg/mL) per mL of cell suspension. Cell debris was removed by ultracentrifugation at 4 °C for 30 min at 13,000 rpm. The supernatant, corresponding to the soluble fraction, was stored at −20 °C for further electrophoresis analysis.

For scale-up production of the recombinant proteins, a total of 12.5 mL of starter culture was used to inoculate 500 mL of auto-induction medium containing 100 µg/mL of kanamycin. The culture was incubated with shaking at 37 °C and 250 rpm for three hours, and then the temperature was adjusted to 28 °C for overnight growth. Finally, 100 mL aliquots of cell suspension were pelleted by centrifugation at 4500 rpm for 30 min at 4 °C. The resulting pellets were washed three times with a 0.8% NaCl solution and then stored at −80 °C.

### 5.4. Purification of the DsbC-CAP Recombinant Protein

For the purification of the recombinant protein, the cell pellets were thawed and lysed directly in binding buffer (20 mM sodium phosphate, 0.5 M NaCl, 20 mM imidazole, pH 7.4) by the addition of 10× concentrated CelLyptic^TM^ B Cell Lysis Reagent supplemented with lysozyme to a final concentration of 300 µg/mL and 1 µL DNase solution (1 mg/mL) per mL of cell suspension. Cell debris was removed by centrifugation at 13,000 rpm for 10 min at 4 °C. The clarified soluble part was filtered through a 0.2 µm nitrocellulose membrane (Merck Millipore) before being loaded onto a metal-chelating affinity column (1 mL HisTrap HP, GE Healthcare, Chicago, IL, USA). Recombinant proteins were loaded into the column, and supernatant flow-through was collected for SDS-PAGE analysis. Next, the column was washed using 10 times the bed volume with binding buffer and 10 bed volumes of wash buffer (20 mM sodium phosphate, 0.5 M NaCl, 50 mM imidazole, pH 7.4). Recombinant proteins were purified with elution buffer (20 mM sodium phosphate, 0.5 M NaCl, 500 mM imidazole, pH 7.4) in 1 mL fractions. Finally, elution fractions from affinity chromatography were pooled, concentrated, and dialysed several times using an Amicon^®^ Ultra Centrifugal Filter with a 50 kDa cutoff (Millipore, Carrigtwohill, Ireland), and a pure DsbC-CAP fusion protein was obtained in 20 mM phosphate buffer saline, pH 7.4 (PBS). The same procedure was applied for the purification of the DsbC tag, which served as a control in the bioassays. Aliquots of the recombinant protein were stored at −20 °C until use.

### 5.5. Electrophoresis Analysis

The supernatant from different expression conditions and purification fractions was denatured using 2× urea sample buffer (8M urea, 1.6% SDS, 0.016% bromophenol blue, 4% β-mercaptoethanol, and 7% glycerol in 50 mM Tris-HCl, pH 6.8). The samples from different expression conditions were analysed in a Tris-Tricine SDS-PAGE with a 16% polyacrylamide resolving gel and a 10% stacking gel on a Mini-Protean II gel system (Bio-Rad, Hercules, CA, USA). All other samples from different purification steps, such as crude extract, exclusion fraction, and elution fractions, were analysed separately in a second Tris-Tricine SDS-PAGE gel.

### 5.6. Mass Spectrometry Analysis

The protein band observed in the elution fraction of nickel affinity chromatography, matching the expected molecular weight, was excised from the SDS-PAGE gel. This excised band was then analysed using mass spectrometry. Proteins were reduced, alkylated, and digested with trypsin (Promega, Madrid, Spain) overnight at 37 °C. The obtained peptides were desalted and concentrated using POROS C18 (Empore 3M, Maplewood, MN, USA). The fractions were applied directly onto a plate using 1 µL of a 5 mg/mL solution of alpha-cyano-4-hydroxycinnamic acid (Sigma, St. Louis, MO, USA) in a mixture of 50% (*v*/*v*) acetonitrile and 5% (*v*/*v*) formic acid. Measurements were completed in positive MS reflector and MS/MS modes using a nano LC-MS coupled to a Sciex TripleTOF 6600 mass spectrometer (Sciex, Darmstadt, Germany). The CalMix5 (Protea Biosciences, Morgantown, WV, USA) standard was used for external calibration of the instrument. The MS spectra’s twenty-five most intense precursor ions were selected for MS/MS analysis. The Protein Pilot Software v. 5.0 (Sciex, Darmstadt, Germany) was used to analyse the raw MS and MS/MS data with the Mascot search engine (MOWSE algorithm). Maximum precursor mass tolerance (MS) was set to 50 ppm, the maximum fragment mass tolerance (MS/MS) was 0.3 Da, and monoisotopic peptide mass values were used as search parameters. The search was conducted against a custom protein database (UniProt) with a taxonomy restriction to *Steinernema* containing the DsbC-CAP protein sequence. The oxidation of methionine and N-Pyro Glu of the N-terminal Q was set as variable modifications, and carboxyamidomethylation of cysteines was set as a fixed modification. Protein identification was only considered when a significant protein homology score was obtained and at least one peptide was fragmented with a significant individual ion score (*p* < 0.05).

### 5.7. Structural Characterisation of the CAP Protein

#### 5.7.1. Predicted Tertiary Structure of Sc-CAP

The tertiary structure of the Sc-CAP protein was predicted with the artificial intelligence system AlphaFold2 ColabFold v1.5.5 [41]. The CAP protein sequence was loaded into ColabFold (https://colab.research.google.com/github/sokrypton/ColabFold/blob/main/AlphaFold2.ipynb, accessed on 20 June 2023), an online platform for protein folding accessible via Google Colab resources. The AlphaFold2 ColabFold v1.5.5 prediction method comprises five steps: multiple sequence alignment (MSA) construction, template search, inference using five models, model ranking based on pLDDT, and relaxation of the predicted structures. The CAP protein structure was predicted by combining the fast homology search of MMseqs2 with AlphaFold2 ColabFold v1.5.5. The AlphaFold2_mmseqs option was selected for MSAs generated by MMseqs2 and relaxed structures predicted using Amber force fields. Molecular representations of the obtained 3D model were prepared using the software UCSF Chimera Version 1.17.3 [42].

#### 5.7.2. Structural Superimposition and Analysis of Phylogeny

The CAP-predicted 3D structural model was used to find the closest homologs through superimposition of models using the Dali server (http://ekhidna2.biocenter.helsinki.fi/dali/, accessed on 12 September 2023), an online tool for protein structure comparison against those in the Protein Data Bank (RCSB PDB).

The amino acid sequence of the CAP protein was used to search for homologs with online pHMMER (https://www.ebi.ac.uk/Tools/hmmer/search/phmmer, accessed on 12 September 2023) against PDB databases with a taxonomy restriction to Nematodes, Platyhelminthes, Fungi, Chordata, and Arthropoda. All obtained closely related structures (Supplementary File S1) were selected to perform multiple comparisons and 3D alignment using the PDBeFold service at the European Bioinformatics Institute website (https://www.ebi.ac.uk/msd-srv/ssm/cgi-bin/ssmserver, accessed on 13 September 2023) [43], and the obtained similarity matrix of Q-scores (Supplementary Table S2) was used to calculate the distance-based matrix and phylogenetic tree using the neighbour-joining method conducted in Mega X [26].

### 5.8. Sterol-Binding Assays

#### 5.8.1. Molecular Docking of Sterols with the Sc-CAP Protein

Docking analyses of various sterols, including cholesterol, palmitate, leukotriene B4, and prostaglandin E2, with the Sc-CAP protein were conducted using AutoDock Vina software version 1.1.2 [44]. Analyses were run with an energy range of 4 and exhaustiveness of 40 to generate the top 10 docking poses. For each sterol/CAP situation, the search spaces were defined in relation to the caveolin binding motif loop (CBM) and the palmitate binding cavity (PBC), as reported in the homologous structure BmVAL-1 from *Brugia malayi* [25]. The PLIP online tool was used to obtain profiles of the best interaction pose for each CAP-ligand situation [45]. Molecular representations of interactions were prepared using the software UCSF Chimera Version 1.17.3 [42] and PyMOL (The PyMOL Molecular Graphics System, Version 2.3.3, Schrödinger, LLC, Portland, OR, USA).

#### 5.8.2. In Vitro Cholesterol-Binding Assay

The sterol-binding capacity of the CAP protein was assessed in vitro by incubation of 80 µg/mL cholesterol with different amounts of the DsbC-CAP recombinant protein in binding buffer (20 mM Tris-HCl, pH 7.5, 30 mM NaCl, and 0.05% Triton X-100) for 1 h at 30 °C. The protein was then separated from the unbound cholesterol using nickel magnetic agarose beads (His Mag Sepharose™ Ni, Merck, Lisbon, Portugal), the liquid above was discarded, and the magnetised beads were rinsed with binding solution. Beads were resuspended in elution buffer (20 mM Tris-HCl, pH 7.5, 500 mM imidazole, 30 mM NaCl, and 0.05% Triton X-100) for 1 min under agitation. After the magnetisation of the beads, the supernatant was collected for the measurement of sterol by cholesterol quantification assay kit CS0005 (Sigma-Aldrich, Lisbon, Portugal). The same procedure was applied to the DsbC tag only in PBS to serve as a negative control. The binding capacity is expressed as the total final amount of cholesterol co-purified with recombinant protein. Three independent assays were conducted under the above experimental conditions, and the data were reported as mean ± S.D.

### 5.9. Insect Physiological Assays

To assess the effect of the CAP protein on the insect immune system, three independent assays were conducted to measure prophenoloxidase (PPO) activity and antimicrobial activity. A total of 60 *Galleria mellonella* larvae cultured in the laboratory were collected, and half of the samples were used as a control and injected with 20 µL of DsbC tag, while the remaining larvae were injected with 20 µL of DsbC-CAP. Both recombinant proteins were injected at a concentration of 1 mg/mL in PBS buffer. The two groups of larvae were stored in Petri dishes and incubated at 25 °C for 18 h.

#### 5.9.1. Prophenoloxidase Assay (PPO)

The prophenoloxidase (PPO) activity was measured in the hemolymph of *G. mellonella* larvae treated with the DsbC-CAP recombinant protein (*n* = 15) and control larvae (*n* = 15).

The hemolymph was collected from the amputated proleg and added into tubes containing cold anticoagulant buffer (10 mM sodium citrate, pH 6.0) in a dilution ratio of 1:20. The hemolymph samples were activated with 1 µL (10 µg/mL) of *E. coli* lipopolysaccharides (Sigma L2637) and incubated for 1 min at 25 °C. The measurement of phenoloxidase began with the addition of 40 µL of diluted hemolymph to 160 µL of 10 mM 3,4-dihydroxyphenylalanine dissolved in 10 mM phosphate buffer, and the increase in absorbance at 490 nm was measured using a microplate absorbance reader spectrophotometer (Thermo Scientific™ Multiskan™ FC, Singapore) for 5 h at 5-minute intervals. Three independent assays were performed under the above experimental conditions, and data were reported as mean ± S.D.

#### 5.9.2. Antimicrobial Response

The antimicrobial activity of the hemolymph was assessed against the Gram-negative bacterium *Escherichia coli* using a microplate method. Briefly, 15 control larvae and 15 CAP-treated larvae were collected, and the hemolymph was extracted from the amputated proleg and diluted 1:1 with an anticoagulant buffer (10 mM sodium citrate, pH 6.0). A total of 30 µL of diluted hemolymph samples were mixed with 160 µL of Luria broth (LB) medium in a CELLSTAR 96-well suspension culture plate (Greiner Bio-One, Frickenhausen, Germany ). *E. coli* was grown overnight in 5 mL of LB medium, and 10 μL of the culture was added to each well. Negative control (NC) contained 10 μL of *E. coli* in 190 μL of LB medium only. The plates were incubated at 37 °C for 6 h, and the optical density at 620 nm was measured to determine the bacterial growth. Three independent assays were performed under the above experimental conditions, and the data were reported as mean ± S.D. The maximum bacterial growth inhibition rates were calculated using the following equation, as described by Guo et al. (2025) [27]:

$$\%Inhibition=\left(1-\frac{OD-OD(0h)}{NC[OD-OD\left(0h\right)]}\right)\times 100$$

### 5.10. Data Analysis and Statistics

All graphs presented in the manuscript were created using GraphPad Prism 8 software. The in vitro sterol-binding assays were conducted in triplicate and repeated three times, and the in vivo injection experiments utilised a total of fifteen biological replicates across three independent assays. Statistical analysis was conducted to compare the control and treated groups using an unpaired t-test. Statistical differences were considered significant at a *p* < 0.05 level. Dead insects during the treatment period were excluded from the assays.

## Figures and Tables

**Table 1:** List of proteins or domains found by loading the Sc-CAP predicted 3D model into the Dali server using multiple structural alignments or 3D superimposition. Z-score is used to sort the neighbours. Similarities are spurious if their Z-score is less than 2. Root mean square deviation is referred to as RMSD. Sequence identity (%id), number of residues (Nres), and number of superposed residues (Lali) are shown. Asterisks indicate protein sequences with multiple domains (*).

Protein/Domain	PDB ID	Species	Function	Z-Score	RMSD	Lali	Nres	%id
BmVAL-1	6ANY	*Brugia malayi*	Sterol binding	29.6	1.7	199	206	43
Na-ASP-2	4NUK	*Necator americanus*	Unknown	28.5	1.6	191	193	41
Na-ASP-1 *	3NT8	*Necator americanus*	Unknown	28.2	2.8	201	401	41
HpVAL-4	5WEE-A	*Heligmosomoides polygyrus bakeri*	Sterol binding	23.5	1.9	183	188	26
HPI	4TPV	*Ancylostoma caninum*	Unknown	21.4	2.0	169	177	30

## Data Availability

The original contributions presented in this study are included in this article and supplementary material. Further inquiries can be directed to the corresponding author.

## Supplementary Materials

The following supporting information can be downloaded at: https://www.mdpi.com/article/10.3390/toxins17070342/s1, Figure S1: Per-residue estimate of confidence on a scale from 0 to 100 (pLDDT) of the Sc-CAP AlphaFold-generated model; Table S1: Q-score structure distance matrix calculated by multiple comparison and 3D alignment of all protein structures using PDBeFold; Table S2: CAP protein identification by LC-MS analysis using a custom protein database with a taxonomy restriction to *Steinernema* containing the DsbC-CAP protein sequence; File S1: List of Sc-CAP closely related structures (PDB files).

## Abbreviations

The following abbreviations are used in this manuscript:

BCA	Biological Control Agent
CAP	Cysteine-rich Secretory Protein/Antigen 5/Pathogenesis-related 1
CBM	Caveolin Binding Motif
DsbC	Disulfide Bond Isomerase C
EPN	Entomopathogenic Nematode
ESPs	Excretory/Secretory Products
IMAC	Immobilized Metal Affinity Chromatography
LB	Luria Broth
OD	Optical Density
PBS	Phosphate Buffer Saline
PBC	Palmitate Binding Cavity
PPO	Prophenoloxidase
Sc-CAP	Steinernema carpocapsae CAP Protein
VAL	Venom Allergen-like Protein
NC	Negative Control
