# Supplementary figures and images for: Immunomodulatory Effect of a Cysteine-Rich Secretory Protein from an Entomopathogenic Nematode with Sterol-Binding Activity
Source: Toxins (Basel). 2025 Jul 5;17(7):342. doi: 10.3390/toxins17070342 (PMC12299418; doi:10.3390/toxins17070342)

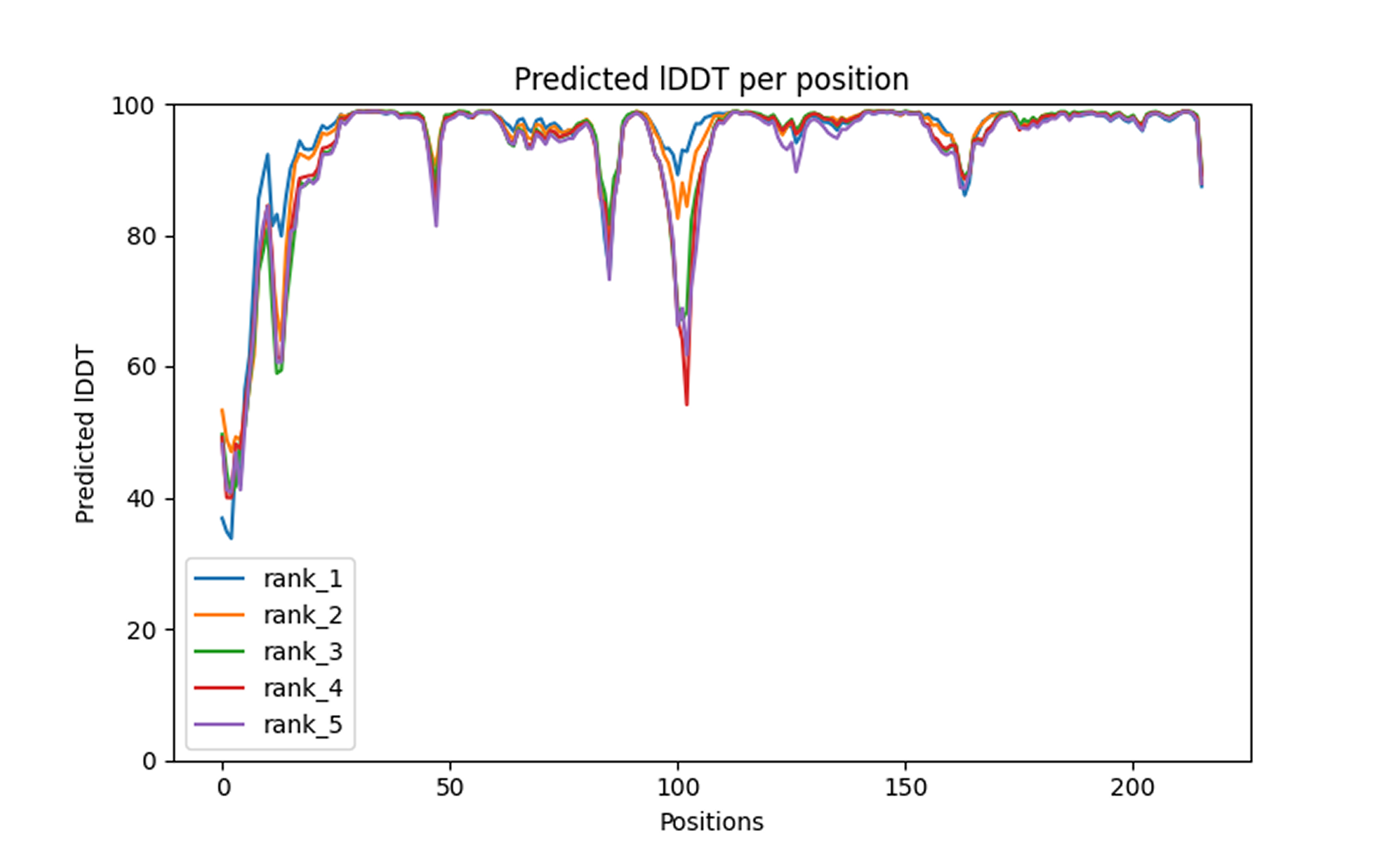

Supplement: Supplementary file 1 [file toxins-17-00342-s001.zip › Supplementary Figure S1.tif]
